# Treadmill Exercise Training Modulates Hepatic Cholesterol Metabolism and Circulating PCSK9 Concentration in High-Fat-Fed Mice

**DOI:** 10.1155/2013/908048

**Published:** 2013-06-19

**Authors:** Shin Wen, Kavita S. Jadhav, David L. Williamson, Todd C. Rideout

**Affiliations:** Department of Exercise and Nutrition Sciences, University at Buffalo, Buffalo, NY 14214, USA

## Abstract

Proprotein convertase subtilisin/kexin type 9 (PCSK9) is a novel biomarker of LDL clearance and a therapeutic target of cardiovascular disease. We examined the effects of aerobic exercise training in modulating PCSK9 abundance and hepatic sterol regulation in high-fat-fed C57BL/6 mice. Mice (*n* = 8) were assigned to a low-fat (LF), high-fat (HF), or an HF with exercise (HF + EX) group for 8 weeks. The HF + EX group was progressively trained 5 days/week on a motorized treadmill. The HF + EX group was protected against body weight (BW) gain and diet-induced dyslipidemia compared with the HF group. The HF + EX group demonstrated an increase in hepatic PCSK9 mRNA (1.9-fold of HF control, *P* < 0.05) and a reduction in plasma PCSK9 (14%) compared with the HF group. Compared with HF mice, HF + EX mice demonstrated reduced hepatic cholesterol (14%) and increased (*P* < 0.05) nuclear SREBP2 protein (1.8-fold of HF group) and LDLr mRNA (1.4-fold of HF group). Plasma PCSK9 concentrations correlated positively with plasma non-HDL-C (*P* = 0.01, *r* = 0.84). Results suggest that treadmill exercise reduces non-HDL cholesterol and differentially modulates hepatic and blood PCSK9 abundance in HF-fed C57BL/6 mice.

## 1. Introduction

Aerobic exercise is consistently associated with favorable shifts in blood triglycerides and HDL-C; however, data from intervention studies [1, 2] and numerous meta-analyses [3–6] also support a less well-characterized and variable LDL-C lowering response to exercise training. Beyond LDL-C lowering, lipoprotein-profiling studies also suggest that high-intensity exercise training may modulate LDL particle number and size distribution patterns [7, 8]. Using a kinetic tracer approach, Ficker et al. recently shed light on the potential mechanism of LDL-C lowering in response to exercise training. They reported increased fractional LDL clearance in both hypercholesterolemic and normolipidemic individuals following a 4-month exercise program consisting of stretching, cycling, and strength training exercises [9]. The degree of LDL-C lowering following exercise is likely dependent on multiple subject-specific factors (baseline lipid status, occurrence and extent of weight loss) and type and rigor of exercise training including schedule, frequency, and volume [3, 10]. 

Blood cholesterol concentrations are maintained within narrow range through complex and coordinated pathways that direct enterohepatic cholesterol absorption and synthesis, intravascular VLDL remodeling, and plasma LDL clearance through the LDL receptor (LDLr) by receptor-mediated endocytosis [11]. Transcriptional regulation of hepatic LDLr expression is classically regulated through sterol regulatory element binding protein 2 (SREBP2), signaled through cellular cholesterol [12]. Understanding of posttranscriptional LDLr regulation was significantly bolstered by the more recent discovery of proprotein convertase subtilisin/kexin type 9 (PCSK9) protein, a serine endoprotease that promotes degradation of the LDLr protein [13]. Function/distribution studies suggest that PCKS9 is synthesized and secreted predominately by the liver and initiates extracellular degradation of membrane-incorporated LDLr following direct binding [14], although intracellular mechanisms may also be involved [15]. PCSK9 shares a common transcriptional regulatory pathway with the LDLr through SREBP2 [16]. As PCSK9 is inversely related to LDL particle clearance, it is considered a potentially important biomarker of cardiovascular disease risk, intimately reflective of hepatic SREBP2 expression and LDLr activity [17]. 

Given the current understanding of the specific role of PCSK9 in regulating hepatic LDLr uptake and the recent report of increased fractional LDL clearance in response to exercise [9], there are emerging questions regarding the potential role of exercise training as a modulator of PCSK9 metabolism. Although plasma PCSK9 concentrations have been examined in response to diet [18–21] and pharmaceutical [22, 23] lipid-lowering therapies, modulation of PCSK9 metabolism has yet to be examined as a potential contributing mechanism underscoring LDL-C reductions and hepatic LDL clearance in response to exercise. Therefore, the objective of this study was to examine the effects of aerobic exercise training in modulating PCSK9 abundance and hepatic sterol regulation in a high-fat-fed C57BL/6 mouse compared with untrained control animals. We hypothesized that aerobic exercise training would limit weight gain and reduce circulating PCSK9 and LDL-C, while consuming a high-fat diet.

## 2. Experimental Approach

The animals used in this experiment were cared for in accordance with the guidelines established by the Institutional Animal Care and Use Committee. All procedures were reviewed and approved by the Animal Care Committee at the University at Buffalo.

### 2.1. Animals, Treadmill Exercise, and Diets

 Two-month old lean male C57BL/6 mice (Taconic Farms, Inc.) were randomly allocated to one of three groups for 8 weeks (Table 1): (1) a low-fat-fed group (LF, 10% fat, Research Diets, Inc.; diet no. D12492i); a high-fat-fed group (HF, 60% fat, Research Diets, Inc.; diet no. D12450Bi); or an HF-fed exercised group (HF + EX) that were trained 5 days/week on a treadmill (26 m/min; Columbus Instruments, Inc.; Exer 3/6). On the first day, animals were placed in the treadmill chamber and allowed to acclimate for 20 min. Each day, animals were given a 3–5 min warm-up period with slow walking speeds. Training began with a pace of 10 m/min, a moderate walk-jog pace, for ~20 min. As mice became increasingly familiar with the treadmill, the velocity and duration were gradually increased until mice were able to run between 25–28 m/min for 45 min. 25–28 m/min is estimated to be 75–80% of maximal oxygen consumption for mice [24]. Generally, both pace and time were increased in an attempt to achieve a ~10% increase per week. Short bursts of compressed air were used to train the mice to continue their pace up to 45 minutes in duration. Mice were constantly monitored while they were on the treadmill. If a mouse required more than 4 bursts of compressed air to continue running within a minute period, the treadmill speed was reduced by ~10%. If a mouse appeared to be injured during the training, it was removed from the treadmill immediately. The HF + EX mice achieved a final grade of 5%, running 45 min/day at 26 m/min by the forty-third session. Body weight and food consumption were monitored weekly throughout the study.

### 2.2. Sample Collection

 All blood and tissue samples were collected two days after the final exercise session to limit acute diet and hormonal influence. Twelve-hour fasting plasma samples were collected by cardiac puncture into heparinized tubes and separated from whole blood by centrifugation at 1,000 ×g for 10 min. Following the two-month treatment period, livers were removed under 3% isoflurane anesthesia and immediately frozen in liquid nitrogen for subsequent analysis. Following tissue removal, the mice were euthanized by transecting the heart and diaphragm while under isoflurane. All tissues were stored at −80°C until further processing and analyses.

### 2.3. Blood Lipid and PCSK9 Analysis

 Plasma total cholesterol, non-HDL-C, and HDL-C were measured by enzymatic kit (BioAssay Systems, EHDL-100) according to the manufacturers' instructions. Commercial ELISA kits were used to assess direct LDL-C (Kamiya Biomedical, KT-21019) and PCSK9 (R&D Systems, MPC900) concentrations.

### 2.4. Hepatic Cholesterol Analysis

Hepatic cholesterol was extracted and analyzed according to our previously published procedures [25, 26]. Approximately 500 mg of pulverized liver was spiked with *α*-cholestane as internal standard and saponified in freshly prepared KOH-methanol at 100°C for 1 h. The nonsaponifiable sterol fraction was extracted with petroleum diethyl ether and dried under N_2_ gas. Sterol fractions were analyzed using a Shimadzu GC-17A gas chromatograph fitted with a flame ionisation detector. A SAC-5 capillary column (30 m × 0.25 mm × 0.25 mm, Supelco, Bellefonte, CA, USA) was used for cholesterol analyses.

### 2.5. Immunoblot Analysis of Hepatic Regulatory Proteins

 Nuclear and cytoplasmic enriched extracts for immunoblot analyses of SREBP1c (Novus Biologicals, NB600-582), SREBP2 (Abcam, ab30682), PCSK9 (Abcam, ab31762), and *β*-actin (Cell Signaling, 8H10D10) were prepared according to our previously published procedures [27]. Briefly, 200 mg of frozen, pulverized liver was homogenized in 10 volumes of CHAPS-containing buffer (40 mM HEPES (pH 7.5), 120 mM NaCl, 1 mM EDTA, 10 mM pyrophosphate, 10 mM *β*-glycerophosphate, 40 mM NaF, 1.5 mM sodium vanadate, 0.3% CHAPS, 0.1 mM PMSF, 1 mM benzamidine, and 1 mM DTT). Supernatant collected following centrifugation at 1,000 ×g for 3 min at 4°C contained the cytoplasmic fraction. The pellet was washed three times with CHAPS buffer, centrifuged at 1,000 ×g for 3 min at 4°C, and then resuspended in 50 *μ*L of lysis buffer and 8.3 *μ*L of 5 M NaCl to lyse the nuclei. The mixture was rotated at 4°C for 1 h and then centrifuged at 12,578 ×g for 15 min at 4°C. The supernatant contained the soluble nuclear fraction.

Hepatic crude membrane for immunoblot analysis of LDLr (Novus Biologicals, NB110-57162) was prepared according to previous reported procedures [28]. Briefly, 200 mg of pulverized, frozen liver was homogenized for 2 min with 1 mL of ice-cold homogenization buffer (10 mM Tris-HCl, 250 mM sucrose, and 2 *μ*g/mL of PMSF; pH 7.5). The homogenate was then centrifuged at 1,000 ×g for 10 min at 4°C to pellet undisrupted cells and nuclei. The collected supernatant was then centrifuged at 100,000 ×g for 1 hour at 4°C. The resulting pellet (containing the crude membrane fraction) was resuspended in 500 *μ*L of resuspension buffer (80 mM NaCl, 2 mM of CaCl_2_, 50 mM Tris-HCl, 1% Triton-X-100, and 2 *μ*g/mL of PMSF; pH 7.5) and stored at −80°C for future analysis. Immunoblots were prepared as previously described [29]. Nuclear and cytoplasmic proteins were normalized to *β*-actin. All blots were quantified using ImageJ (National Institutes of Health, Bethesda, MD, USA).

### 2.6. Hepatic RNA Preparation and Real-Time RT-PCR

 Total RNA was isolated from whole liver tissue using TRIzol reagent (Ambion, AM9738). RNA concentration and integrity were determined with spectrophotometry (260 nm) and agarose gel electrophoresis, respectively. RNA preparation and real-time RT-PCR were conducted using a one-step QuantiTect SYBR Green RT-PCR kit (Qiagen, 204154) on a Biorad MyiQ real-time PCR system according to previously established protocols [29]. Gene expression was analyzed using the 2(-delta delta Ct) method [30]. Sequences of sense and antisense primers for target and housekeeping genes were based on previously published sequences for PCSK9 [31], LDLr [32], and *β*-actin [33].

### 2.7. Statistical Analyses

Whole-body growth and blood lipid responses between LF, HF and HF + EX groups were compared using Dunnett's test. Comparisons between HF, and HF + EX groups were conducted using a paired *t*-test. The association between plasma PCSK9 and non-HDL cholesterol was assessed with Pearson's product moment correlation coefficients. Data were analyzed with SPSS 16 for Mac (SPSS Inc, Chicago, IL, USA). Data are presented as mean ± SEM. All results are the means from 8 animals unless otherwise stated. Differences were considered significant at *P* ≤ 0.05.

## 3. Results

### 3.1. Body Weight and Food Intake

**Figure 1:**
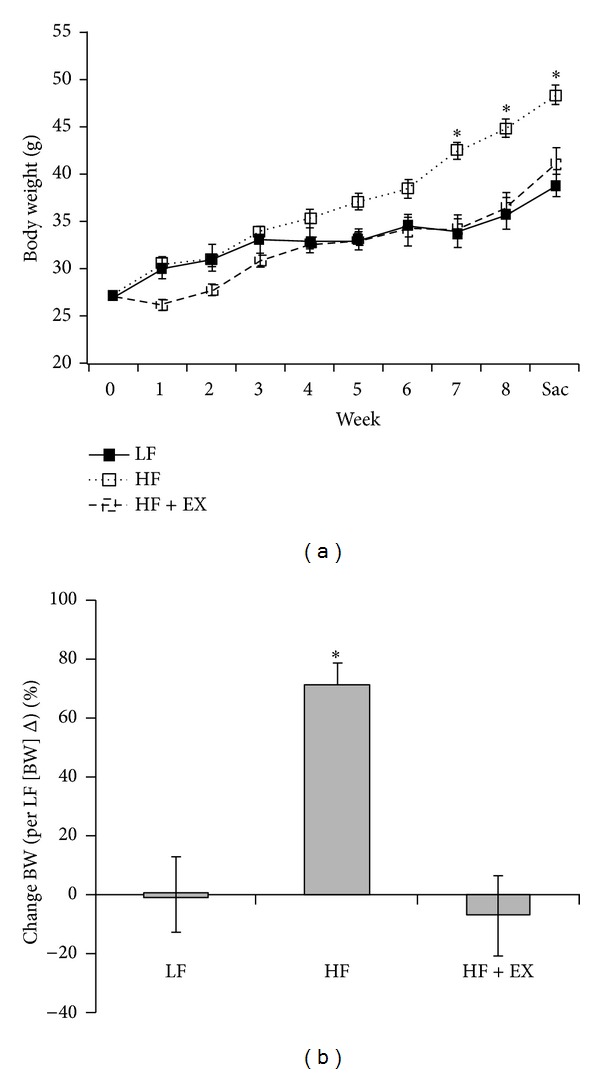
Time course (a) and % change (normalized to LF absolute weight change for duration of study) (b) of body weight gain in C57BL/6 mice assigned to a low-fat (LF), high-fat (HF), or a high-fat/exercise (HF + EX) group for 8 weeks. Values are mean ± SE, *n* = 8; ∗ denotes a significant difference from LF group (*P* < 0.05).

 Mice that consumed an HF diet increased body weight (BW) gain (*P* < 0.05) when compared with the LF or the HF + EX groups, expressed as endpoint BW (Table 2), BW over time (Figure 1(a)), and as a percent of absolute BW gain over time (Figure 1(b)). Changes in BW were independent of feed intake as no difference (*P* > 0.05) was observed between the LF, HF, and HF + EX groups (Table 2). The HF group also showed increased liver weight gain (*P* < 0.05) when compared to the LF and the HF + EX groups (Table 2).

### 3.2. Lipid Response

**Figure 2:**
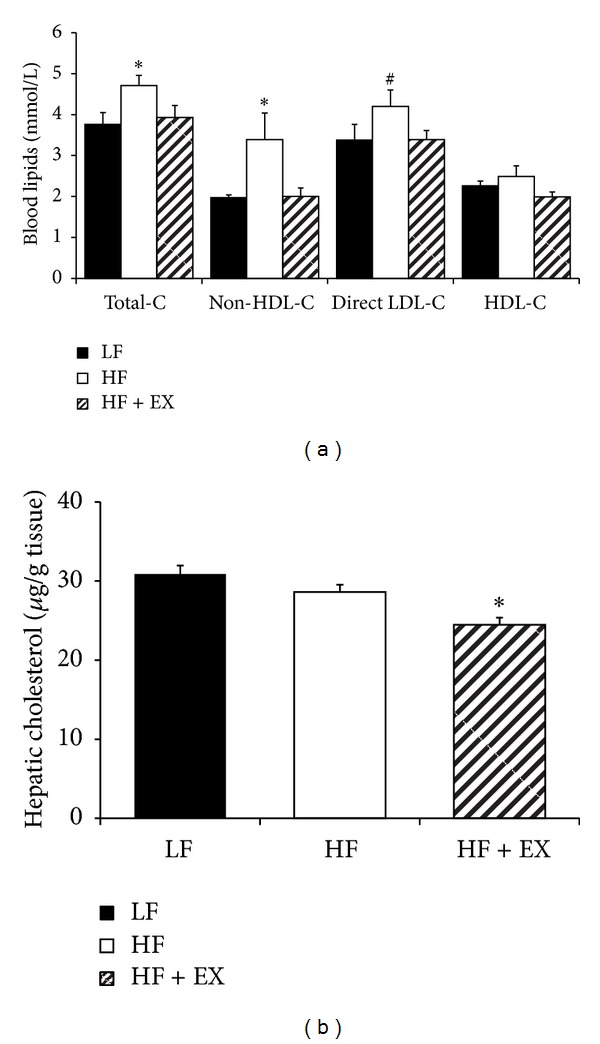
Blood lipid (mmol/L) and hepatic cholesterol (mg/g tissue) response in C57BL/6 mice assigned to a low-fat (LF), high-fat (HF), or a high-fat/exercise (HF + EX) group for 8 weeks. (a) Blood lipids including total cholesterol; non-HDL-C, LDL-C, and HDL-C (mmol/L); and (b) hepatic cholesterol (*μ*g/g tissue). Values are mean ± SE, *n* = 8; ∗ denotes a significant difference from LF (*P* < 0.05); # denotes a difference from LF (*P* = 0.07).

Although HF feeding increased (*P* < 0.05) blood total, non-HDL, and LDL-C (25, 70, and 24%, resp.) compared with the LF-fed animals, exercise training normalized HF-diet induced dyslipidemia to LF-control levels (Figure 2(a)). Exercise training (HF + EX) also reduced (*P* < 0.05) hepatic total cholesterol concentration (14%) compared with the LF group with no difference (*P* > 0.05) observed between the LF and HF animals (Figure 2(b)). The LF feeding group was included to establish effects of high-fat feeding on body weight and blood and tissue lipid responses and as such was not included in the mechanistic gene and protein expression analysis.

### 3.3. Gene and Protein Expression

**Figure 3:**
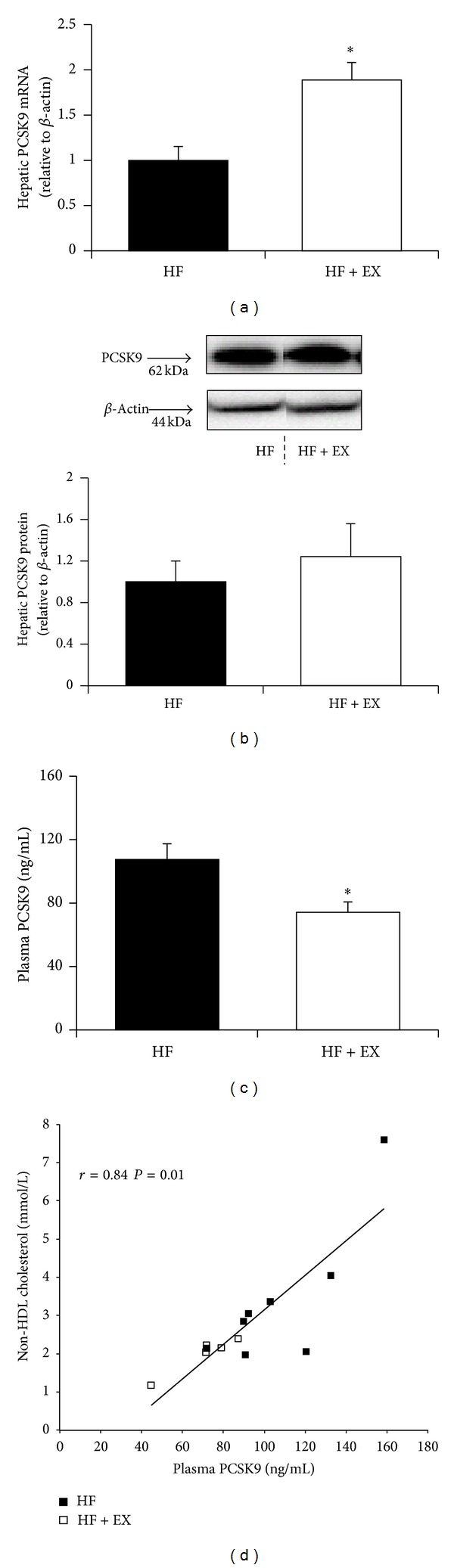
PCSK9 response in C57BL/6 mice assigned to a high-fat (HF) or a high-fat/exercise (HF + EX) group for 8 weeks. (a) Hepatic PCSK9 mRNA expression; (b) hepatic PCSK9 protein abundance; (c) plasma PCSK9 (ng/mL); and (d) Pearson's product-moment correlation (*r*) between plasma PCSK9 and non-HDL cholesterol concentrations. All expression data are normalized to *β*-actin and expressed relative to the HF group; values are mean ± SE, *n* = 8; ∗ denotes a significant difference (*P* < 0.05). Representative PCSK9 protein expression blots were cropped from the same membrane.

**Figure 4:**
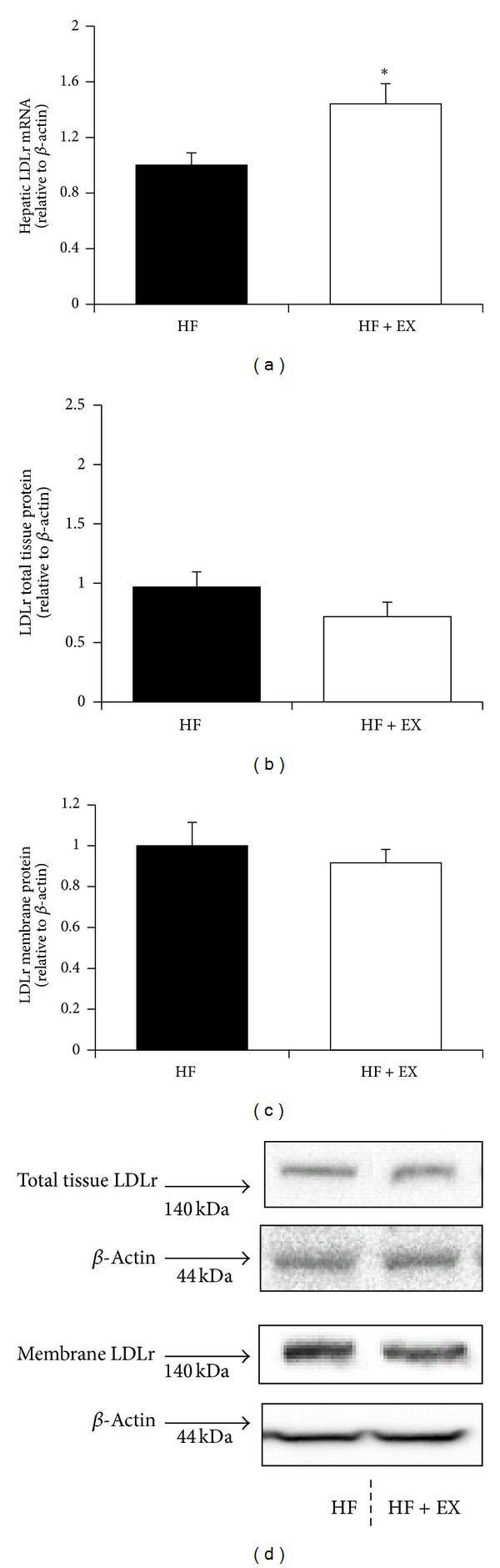
Hepatic LDLr expression in C57BL/6 mice assigned to a high-fat (HF) or a high-fat/exercise (HF + EX) group for 8 weeks. (a) LDLr mRNA expression; (b) hepatic total tissue LDLr protein abundance; (c) hepatic membrane-bound LDLr protein abundance; and (d) representative immunoblots. All data are normalized to *β*-actin and expressed relative to the HF group; values are mean ± SE, *n* = 8; ∗ denotes a significant difference (*P* < 0.05). Representative LDLr protein expression blots were cropped from the same membrane.

 Compared with the HF animals, the HF + EX group demonstrated increased (*P* < 0.05) hepatic PCSK9 mRNA expression (1.9-fold of HF control) with no change observed (*P* > 0.05) in PCSK9 protein abundance (Figures 3(a) and 3(b)). The HF + EX animals exhibited a 31% reduction (*P* < 0.05) in plasma PCSK9 concentrations compared with the HF group (Figure 3(c)). Plasma PCSK9 correlated positively with plasma LDL-C (*P* = 0.01, *r* = 0.82) and non-HDL-C (*P* = 0.01, *r* = 0.84, Figure 3(d)). Although LDLr mRNA expression was increased (*P* < 0.05, 1.4-fold of HF control) in the HF + EX group compared with the HF animals (Figure 4(a)), we observed no change (*P* > 0.05) in LDLr protein abundance in hepatic total tissue or membrane extracts between trained and untrained animals (Figures 4(b), and 4(c)). 

**Figure 5:**
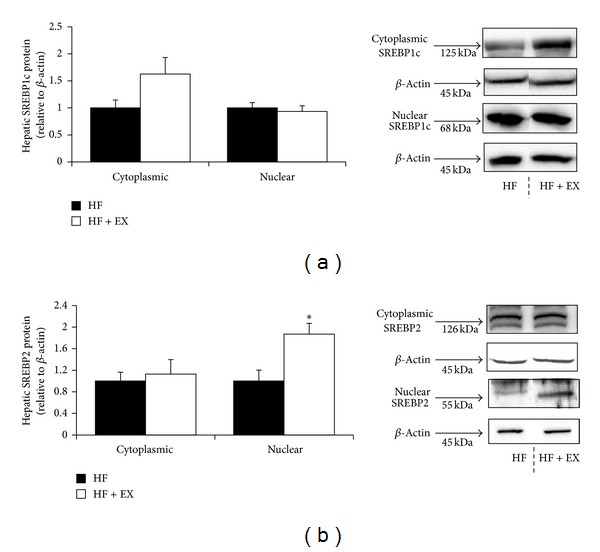
Hepatic SREBP1c and SREBP2 protein abundance in C57BL/6 mice assigned to a high-fat (HF) or a high-fat/exercise (HF + EX) group for 8 weeks. (a) Cytosolic and nuclear SREBP1c abundance. (b) Cytosolic and nuclear SREBP2 abundance. All data are normalized to *β*-actin and expressed relative to the HF group; values are mean ± SE, *n* = 8; ∗ denotes a significant difference (*P* < 0.05). Representative SREBP protein expression blots were cropped from the same membrane.

SREBP1c protein abundance (cytosolic and nuclear) did not differ between the HF and HF + EX groups (Figure 5(a)); however, the HF + EX group demonstrated an increase in nuclear SREBP2 protein abundance (*P* < 0.05, 1.8-fold of HF control) compared with the HF animals (Figure 5(b)). 

## 4. Discussion

Findings from this study suggest that exercise training protects against diet-induced dyslipidemia and differentially modulates the hepatic expression and circulating concentrations of PCSK9. HF + EX animals exhibited an increase (*P* < 0.05) in hepatic PCSK9 mRNA expression (1.9 fold of HF control) but a reduction in circulating plasma PCSK9 concentration (−31%) compared with the HF-fed untrained group. Exercise training also reduced (*P* < 0.05) hepatic cholesterol concentration (14%) and increased the expression of LDLr mRNA (1.4-fold of HF control) and nuclear SREBP2 protein (1.8-fold of HF control).

Our results support previous reports of reductions in plasma cholesterol (total, non-HDL, and/or LDL-C) in response to aerobic exercise training in a variety of rodent models including LDLr^−/−^ [34–37], db/db [38], MC4R^−/−^ mice [39], and male Wistar rats [40]. Alternatively, exercise training failed to increase HDL-C concentrations in our C57BL/6 mouse model, an effect, that is, most often associated with aerobic exercise training in humans [41] but has been shown to be quite variable in rodents [40, 42–44]. This nonresponse of HDL-C is likely model specific as C57BL/6 mice carry the majority (~70%) of plasma cholesterol in the HDL fraction and exhibit modified HDL metabolism characterized by an absence of cholesterol-ester transfer protein and a resistance to diet-induced atherosclerosis [45]. Differences in HDL metabolism in wild-type mice have been suggested to mask the potential protective effects of exercise on HDL concentration [46]. 

To our knowledge, this is the first study to demonstrate a reduction in plasma PCSK9 concentration in response to exercise training. HF + EX mice also exhibited a coordinate upregulation of hepatic PCSK9 and LDLr mRNA (Figures 3(a) and 4(a), resp.), likely the result of a reduction in hepatic cholesterol (Figure 2(b)) and subsequent induction of nuclear SREBP2 protein abundance (Figure 5(b)). In response to reduced cellular cholesterol levels, both PCSK9 and the LDLr are transcriptionally upregulated following SREBP2 cleavage at the endoplasmic reticulum and subsequent nuclear translocation [16, 31]. Although only a limited amount of work has examined hepatic cholesterol metabolism in trained animals, previous studies support our observed reduction in hepatic cholesterol [34] and increased LDLr transcription [47, 48] in exercise-trained C57BL/6 mice. Exercise has been shown to increase SREBP2 mRNA expression in skeletal muscle [49], but we are not aware of any study examining hepatic SREBP2 expression in response to an exercise intervention.

The reduction in plasma PCSK9 was positively correlated with concurrent reductions in plasma LDL-C and non-HDL-C (Figure 3(d)). This correlation may suggest that the reduction in LDL-C in the exercised animals is associated with a lower LDLr turnover and enhanced LDL-C clearance. However, given the recent report from Kosenko et al. suggesting that circulating PCSK9 directly binds to LDL plasma particles, it is equally likely that our observed reductions in PCSK9 are merely reflective of lower LDL in the exercise-trained animals [50]. This may also explain our observed differential response between liver and plasma PCSK9 protein abundance in the exercised animals. Even though LDLr mRNA was elevated in response to exercise training, we failed to detect any difference in hepatic LDLr protein abundance in whole tissue or membrane extracts between trained and untrained animals. Although this finding is unexpected, the action of PCSK9 on LDLr expression and activity in extrahepatic tissues cannot be ruled out as contributing to the observed cholesterol reductions in the exercised animals [51]. Furthermore, we did not examine hepatic VLDL receptor expression, an additional target of PCSK9 that regulates plasma non-HDL cholesterol concentration [52].

Though the precise molecular mechanism(s) underlying exercised-induced modulation of PCSK9 may not beclear, our data suggests that the reduction in blood PCSK9 involves a posttranscriptional and/or translational event(s), given that no change in hepatic PCSK9 protein was observed between the HF and HF + EX groups. While the intramolecular processing events that govern the maturation, secretion, and eventual proteolytic inactivation of PCSK9 have not been fully elucidated, it is clear that nutritional and hormonal signals tightly regulate hepatic expression and plasma concentrations [31, 53]. In this regard, it is interesting to speculate that the protective effects of exercise against weight gain in the present study were related to the observed reduction in PCSK9 concentrations. Resistin, an adipose tissue-derived adipokine, that is, increased in obese rodents, has recently been shown to inhibit hepatic LDL clearance by increasing PCSK9 expression [54]. As weight loss through exercise intervention reduces circulating resistin concentrations [55, 56], reductions in fat mass and adipose-derived resistin secretion may underlie the observed reduction in PCSK9 concentration. 

This study has several limitations. First, it is not known whether the reduced plasma PCSK9 in the HF + EX mice was a cause or a consequence of changes in plasma LDL-C. Without the benefit of a PCSK9-deficient mouse, our results cannot demonstrate a direct link between PCSK9 and LDL-C reductions in response to exercise. Second, the mature PCSK9 protein is composed of a prodomain, a catalytic domain that binds to the EGF-A domain of the LDLr and a C-terminal domain that interacts with cell-surface proteins. Previous work suggests that PCSK9 activity is regulated by specific autocatalytic processing events within the endoplasmic reticulum and is inactivated following cleavage by furin, another protein convertase [14]. The commercial PCSK9 antibody used in these experiments was specific to the mature form of PCSK9; hence, we were not able to examine potential changes in pro-PCSK9 abundance in response to exercise training. Also, it is not clear if the total PCSK9 mass abundance in liver and blood is reflective of its activity. 

## 5. Conclusions

In summary, our results suggest that exercise training reduces blood non-HDL cholesterol and PCSK9 concentrations and enhances the hepatic mRNA expression of SREBP2, LDLr, and PCSK9. Additional mechanistic studies are required to directly link exercise-induced lipid lowering with reduced PCSK9 activity. 

## Figures and Tables

**Table 1:** Formulation of low-fat and high-fat diets fed for C57BL/6 mice.

Ingredient^1^	Diets	
	Low fat^2^	High fat^3^
Casein	19.0	25.8
Corn starch	29.9	0.0
Maltodextrin	3.3	16.2
Sucrose	33.2	8.9
Cellulose	4.7	6.5
Soybean oil	2.4	3.2
Lard	1.9	31.7
Other	5.7	7.8
Total	**100**	**100**
Macronutrient profile (% energy)		
Protein	20	20
Carbohydrate	70	20
Fat	10	60

^1^% composition; ^2^Research Diets, Inc.; diet no. D12492i; ^3^Research diets, Inc.; Diet no. D12450Bi.

**Table 2:** Body weight, liver weight, average feed intake, and total distance run in C57BL/6 mice assigned to a low-fat (LF), high-fat (HF), or a high-fat/exercise (HF + EX) group for 8 weeks.

Parameter	LF^1^	HF^1^	HF + EX^1^
Body weight (g)	38.8 ± 1.3	49.8 ± 0.9*	41.1 ± 1.0
Liver weight (mg)	160.1 ± 15.5	249.5 ± 25.4*	136.3 ± 7.3*
Average feed intake (g/mouse/day)	2.5 ± 0.1	2.3 ± 0.1	2.2 ± 0.0
Total distance run (km)	N/A	N/A	39.4 ± 0.1

^1^LF: low fat; HF: high fat; HF + EX: high fat + exercise. Values are mean ± SE, *n* = 8; ∗denotes a significant difference from LF group (*P* < 0.05).
